# Colony-stimulating factor 1 receptor inhibition prevents microglial plaque association and improves cognition in 3xTg-AD mice

**DOI:** 10.1186/s12974-015-0366-9

**Published:** 2015-08-01

**Authors:** Nabil N. Dagher, Allison R. Najafi, Kara M. Neely Kayala, Monica R. P. Elmore, Terra E. White, Rodrigo Medeiros, Brian L. West, Kim N. Green

**Affiliations:** Department of Neurobiology and Behavior, Institute for Memory Impairments and Neurological Disorders, University of California, 3208 Biological Sciences III, Irvine, CA 92697-4545 USA; Plexxikon Inc., Berkeley, CA USA

**Keywords:** Alzheimer’s disease, Neuroinflammation, Cognition, Therapeutics

## Abstract

**Background:**

Microglia are dependent upon colony-stimulating factor 1 receptor (CSF1R) signaling for their survival in the adult brain, with administration of the dual CSF1R/c-kit inhibitor PLX3397 leading to the near-complete elimination of all microglia brainwide. Here, we determined the dose-dependent effects of a specific CSF1R inhibitor (PLX5622) on microglia in both wild-type and the 3xTg-AD mouse model of Alzheimer’s disease.

**Methods:**

Wild-type mice were treated with PLX5622 for up to 21 days, and the effects on microglial numbers were assessed. 3xTg-AD mice were treated with PLX5622 for 6 or 12 weeks and effects on microglial numbers and pathology subsequently assessed.

**Results:**

High doses of CSF1R inhibitor eliminate most microglia from the brain, but a 75 % lower-dose results in sustained elimination of ~30 % of microglia in both wild-type and 3xTg-AD mice. No behavioral or cognitive deficits were found in mice either depleted of microglia or treated with lower CSF1R inhibitor concentrations. Aged 3xTg-AD mice treated for 6 or 12 weeks with lower levels of PLX5622 resulted in improved learning and memory. Aβ levels and plaque loads were not altered, but microglia in treated mice no longer associated with plaques, revealing a role for the CSF1R in the microglial reaction to plaques, as well as in mediating cognitive deficits.

**Conclusions:**

We find that inhibition of CSF1R alone is sufficient to eliminate microglia and that sustained microglial elimination is concentration-dependent. Inhibition of the CSF1R at lower levels in 3xTg-AD mice prevents microglial association with plaques and improves cognition.

## Introduction

Microglia are the primary immune cell of the central nervous system (CNS), comprising ~12 % of all cells found there. They are found ubiquitously throughout the CNS and function to detect pathogens and insults and respond to them through complicated physical and chemical remodeling processes. While microglia are crucial for the protection of the CNS from pathogens, as well as in clearing up cellular debris following cell death or minor injuries, they are well studied for their roles in neurodegenerative diseases and CNS injuries, such as traumatic brain injuries and stroke [1, 2]. It is generally thought that microglia mount an initial beneficial response, helping to limit damage from injury [3], or phagocytosing aggregating toxic peptides such as Aβ [4] and perhaps limiting plaque formation. However, these initial responses evolve into chronic inflammatory processes that may never resolve and can be damaging to the local brain environment, thus contributing to the disease/injury process itself [5]. Hence, finding ways to manipulate microglial function and numbers may offer a way to treat CNS disorders. To that end, we recently discovered that treatment of adult mice with PLX3397, the dual colony-stimulating factor 1 receptor (CSF1R) and c-kit kinase inhibitor, leads to the near-complete elimination of all microglia from the CNS within 7–21 days [6]. Furthermore, microglia remain eliminated for the duration of treatment, allowing for indefinite microglial elimination from the adult CNS. As CSF1R knockout mice are born without microglia [7, 8], and mice lacking either of its two substrates, CSF1 [9] or IL-34 [10], also have reduced microglial densities; it suggests that microglia are fully dependent upon CSF1R signaling for their survival. Here, we describe the effects of a specific CSF1R inhibitor, PLX5622, on microglial homeostasis in the adult brain. PLX5622 is a potent inhibitor of CSF1R tyrosine kinase activity (KI = 5.9 nM) with at least 50-fold selectivity over 4 related kinases, and over 100-fold selectivity against a panel of 230 kinases [11–14]. As prolonged microglial elimination may not be translatable to humans for the extended duration of a neurodegenerative disease, we have explored the effects of lower, and more clinically relevant, drug exposures on microglial phenotypes and animal cognition/behavior in both adult healthy mice and a mouse model of Alzheimer’s disease.

## Methods

### Compounds

PLX5622 was provided by Plexxikon Inc. and formulated in AIN-76A standard chow by Research Diets Inc. at the doses indicated in the text.

### Animal treatments

All rodent experiments were performed in accordance with animal protocols approved by the Institutional Animal Care and Use Committee at the University of California, Irvine (UCI). *LPS treatment*: LPS (from *Escherichia coli* 055:B5; Sigma) was dissolved in phosphate buffered saline (PBS) at a concentration of 0.1 mg/ml and administered intraperitoneally (IP) at a dose of 0.5 mg/kg body weight. Following any treatments, mice were sacrificed and brains isolated. One-half of the brain was fixed in 4 % paraformaldehyde and the other half was snap frozen on dry ice and stored at −80 °C until analysis.

### Thioflavin S staining

Brain sections were incubated in 0.5 % thioflavin S solution in 50 % ethanol for 10 min, rinsed twice in 50 % ethanol, then rinsed twice in water. Sections were visualized with a confocal microscope. Average plaque number, size, and percentage distribution of size were obtained using Bitplane Imaris 7.4 software.

### Confocal microscopy

Fluorescent immunolabeling followed a standard indirect technique (primary antibody followed by fluorescent secondary antibody) as described in [15]. Cell counts and sizes were obtained by scanning regions at 10× at comparable sections in each animal, followed by automatic analyses using Bitplane Imaris 7.4. Acid pretreatments were used for 6e10 detection. The following antibodies were used: anti-IBA1 (1:1000; Wako), anti-GFAP (1:1000; Dako), anti-6e10 (1:1000; Chemicon), and anti-S100 (1:1000; Abcam). Stained tissue was mounted on slides and cover slipped with Dapi Fluoromount-G (SouthernBiotech).

### Aβ ELISA

Aß_1–40_ and Aß_1–42_ were measured using a sensitive sandwich ELISA system as previously described [16].

### mRNA extraction and real-time PCR

Total mRNA was extracted from frozen half brains, cDNA was synthesized, and real-time PCR (RT-PCR) was performed with commercially available kits for *TNFα* (F, 5′-GGTGCCTATGTCTCAGCCTCTT; R, 5′-GCCATAGAACTGATGAGAGGGAG) and *IL1β* (F, 5′-TGGACCTTCCAGGATGAGGACA; R, 5′- GTTCATCTCGGAGCCTGTAGTG). CT values were normalized to GAPDH and expressed as a percent of control. Two-month-old wild-type mice were treated with PLX5622 (1200 mg/kg chow; *n* = 4) or vehicle (*n* = 4) for 14 days. On day 14, half of the mice in each dietary group were administered with either LPS (0.5 mg/kg) or an equivalent volume of PBS via IP injection (*n* = 4 per group). Mice were euthanized 6 h after injection, perfused with PBS, and their brains were collected and snap frozen with dry ice for RNA extraction.

### Behavioral testing

*Open field*: Open-field testing was employed as a measure of anxiety as well as motor ability. Mice were placed in an opaque white box (33.7 cm L × 27.3 cm W × 21.6 cm H) for 5 min while their behavior was video-recorded. The amount of time spent in the center versus the perimeter of the box and motor readouts (distance moved and velocity) were obtained. *Barnes maze*: Mice were tested in the Barnes maze (table diameter 122 cm, 40 holes with diameter 4.8 cm, elevated 140 cm above the ground) for 5 days (acquisition, 4 days, 2 trials/day, maximum 120 s/trial, 15 min intertrial interval; 24-hr probe, 1 day, 1 trial, maximum of 120 s), as a measure of spatial learning and memory. Latency to find the target and enter the target box (i.e., escape latency) was recorded live each day of acquisition, while latency to find the target and number of errors prior to finding the target was recorded live on the probe day. *Morris water maze*: Hidden platform Morris water maze (MWM) training and testing were conducted as described previously [17]. Novel object and place recognition tasks: Novel object and place recognition training and testing were conducted as described previously [18].

### Chemotaxis assay

Aβ42 was oligomerized as previously described [19]. One micromolar of this stock was added to BV2 cell cultures 24 h before the assay to create Aβ-stimulated enriched media. This media was used as a chemoattractant in the ChemoTx® Chemotaxis System. BV2 cells were treated either 15 min or 24 h before the assay with 1- or 10 μM of PLX5622 in DMSO, and 50,000 cells were added to each well on the assay plate. Cells were allowed to migrate for 3 h. Migrated cells were stained with H+E and counted.

### Statistics

Appropriate statistical analyses were carried out to determine significance between groups using unpaired Student’s *t* test for comparisons between two groups, one-way ANOVA for multiple comparisons, with Newman-Keuls post hoc multiple comparison test. Multiple-day behavioral data and MSD® Multi-Spot Assay data were analyzed using a two-way ANOVA (treatment x day of testing and diet x injection, respectively) using the MIXED procedure of the Statistical Analysis Systems software (SAS Institute Inc.). For behavioral data, “mouse” was a random effect and “day of testing” was a repeated measure. Post hoc paired contrasts were used to examine biologically relevant interactions from the two-way ANOVA.

## Results

### The selective CSF1R inhibitor PLX5622 reduces microglial numbers in the adult brain

**Fig. 1:**
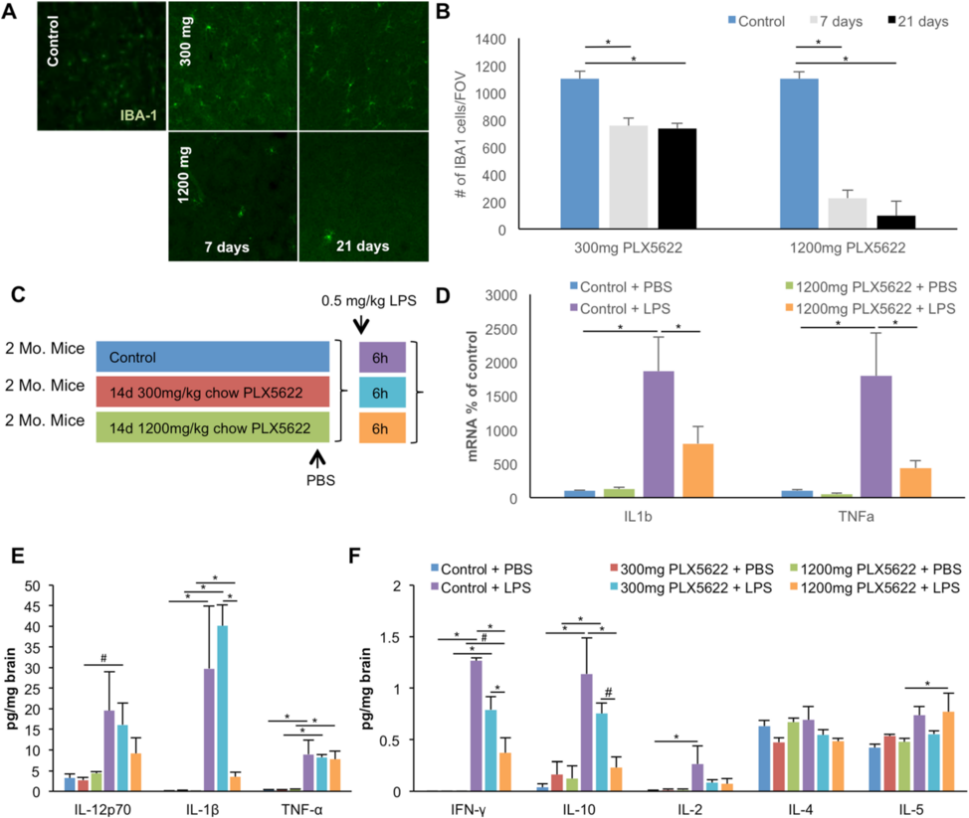
CSF1R inhibition eliminates microglia from the adult brain. Two-month-old wild-type mice were treated with vehicle, 300- or 1200 mg/kg PLX5622 for 7 or 21 days. **a** IBA1 immunofluorescent staining was performed on vehicle, 300 and 1200 mg/kg treated animals; representative 10× confocal images are shown for 7 and 21 days of treatment. **b** Quantification of the number of IBA1^+^ cells for all groups was performed using IMARIS software. **c**–**f** 2 month-old wild-type mice were treated with 300 or 1200 mg/kg PLX5622 or vehicle for 14 days, and LPS or PBS was then administered via IP (0.5 mg/kg). mRNA for *TNFα* and *IL-1β* was measured and normalized to GAPDH in control and PLX5622-treated mice injected with PBS or LPS, showing a marked increase in both inflammatory markers in control groups injected with LPS and a dampened response to LPS in PLX5622-treated groups (**d**). **e**, **f** Inflammatory markers were measured via MSD® Multi-Spot Assay, revealing increases in nearly all markers with LPS injection; treatment with 1200 mg/kg PLX5622 treatment lowered the LPS-induced elevated levels of IFNγ, IL-10, and IL-1β. Treatment with 300 mg/kg PLX5622 had no significant effect on elevated levels of LPS-induced elevated levels of markers. *Indicates significance (*p* > 0.05), # indicates a statistical trend (*p* < 0.1), via two-way ANOVA with post hoc paired contrasts. Error bars indicate SEM

To determine the effects of specific CSF1R inhibition on microglial numbers in the adult brain, 2-month-old wild-type mice were treated with vehicle or PLX5622 at 300- or 1200 mg/kg in chow for 7 or 21 days (*n* = 3 per group). Mice were sacrificed and their brains fixed and sectioned and then stained with the microglial marker IBA1 (Fig. 1a, quantified in B). Confocal images of the cortex were taken with a 10× objective, and automated cell counts were then performed. Quantification revealed that 7 and 21 days of treatment with 300 mg/kg chow caused a sustained ~30 % reduction in microglia numbers. However, treatment with 1200 mg/kg chow led to rapid and robust reductions in microglial numbers, with an 80 % reduction seen within 7 days of treatment. Treatment with either PLX5622 or PLX3397 eliminates microglia, but the former is able to do so without also inhibiting c-kit. These results show that specific inhibition of the CSF1R alone is sufficient for elimination of microglia from the adult CNS. Additionally, we can tightly control the amount of microglial elimination by changing the CSF1R inhibitor dose—300 mg/kg chow results in sustained 30 % elimination, and thus, elimination is not an all or nothing event but something that can be modulated.

We next explored the response of the microglia-depleted brain to systemically administered LPS. Two-month-old wild-type mice were treated with PLX5622 (300 or 1200 mg/kg chow) or vehicle for 14 days, and 0.5 mg/kg LPS was then administered (IP; *n* = 4 per group). Mice were sacrificed 6 h later, and mRNA and protein levels of inflammatory markers were measured from brain tissue via real-time PCR (normalized to the housekeeping gene *GAPDH*) and MSD® Multi-Spot Assay, respectively (Fig. 1c–f). As expected, LPS treatment robustly increased RNA levels of both *TNFα* and *IL-1β* message in microglia-intact animals and increased protein levels of nearly all inflammatory markers examined, with the exception of IL-4. Levels of TNFα and IL-1β mRNA in response to LPS in the 1200 mg/kg PLX5662-treated mice were significantly diminished (Fig. 1d), and protein levels of IFN-γ, IL-10, and IL-1β in response to LPS were also decreased with 1200 mg/kg PLX5622 treatment; however, no changes in inflammatory markers were seen with 300 mg/kg PLX5622 treatment (Fig. 1e, f). Interestingly, although the mRNA levels for TNFα decreased with 1200 mg/kg PLX5622 treatment in response to LPS, TNFα protein levels were not reduced. Although apparently contradictory, these data likely reflect the ability of TNFα to cross the BBB from the periphery [20]. Indeed, plasma TNFα levels in these samples were highly and significantly elevated in all LPS-injected groups (data not shown).

### Microglial depletion does not affect behavior or learning and memory

**Fig. 2:**
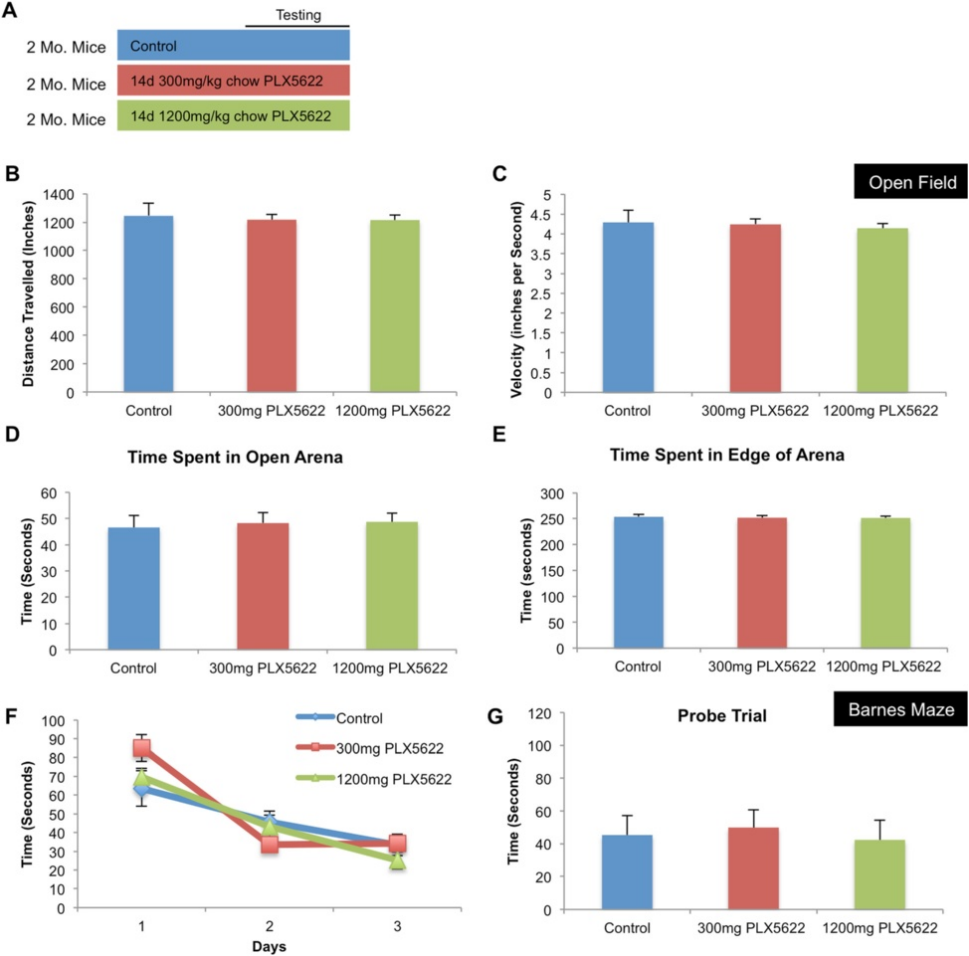
CSF1R inhibition does not alter cognition or behavior in adult mice. **a** Two-month-old wild-type mice were treated with either vehicle, 300 or 1200 mg/kg PLX5622 for 14 days, and behavioral analyses were conducted using an open-field test and Barnes maze. **b**–**e** No differences were measured across groups in the open-field test in distance traveled, velocity, time spent in open arena, or time spent in edge of arena. **f**–**g** No differences were measured across groups in the Barnes maze in acquisition of time to find escape hole or in probe trial. Error bars indicate SEM

Two-month-old wild-type mice were treated with either vehicle or 300- or 1200 mg/kg chow PLX5622 for 14 days, and behavioral analyses were then conducted (*n* = 10 per group) (Fig. 2a–g). Mice were first tested in open field analyses. No differences were seen in mice with either dose of PLX5622 in the distance traveled (Fig. 2b), velocity (Fig. 2c), time spent in the open area (Fig. 2d), or time spent in the edge of the arena (Fig. 2e), compared to vehicle-treated mice. Next, mice were tested on Barnes maze—a hippocampal-dependent learning and memory task. Again, no differences were found between any of the groups on acquisition of the time to find the escape hole (Fig. 2f) or in the probe trial conducted 24 h after the last training session (Fig. 2g). Thus, depletion of microglia with PLX5622 does not induce any deficits in these particular tasks, in agreement with our previous data [6].

### Microglial elimination with PLX5622 followed by drug removal results in rapid repopulation of the CNS

**Fig. 3:**
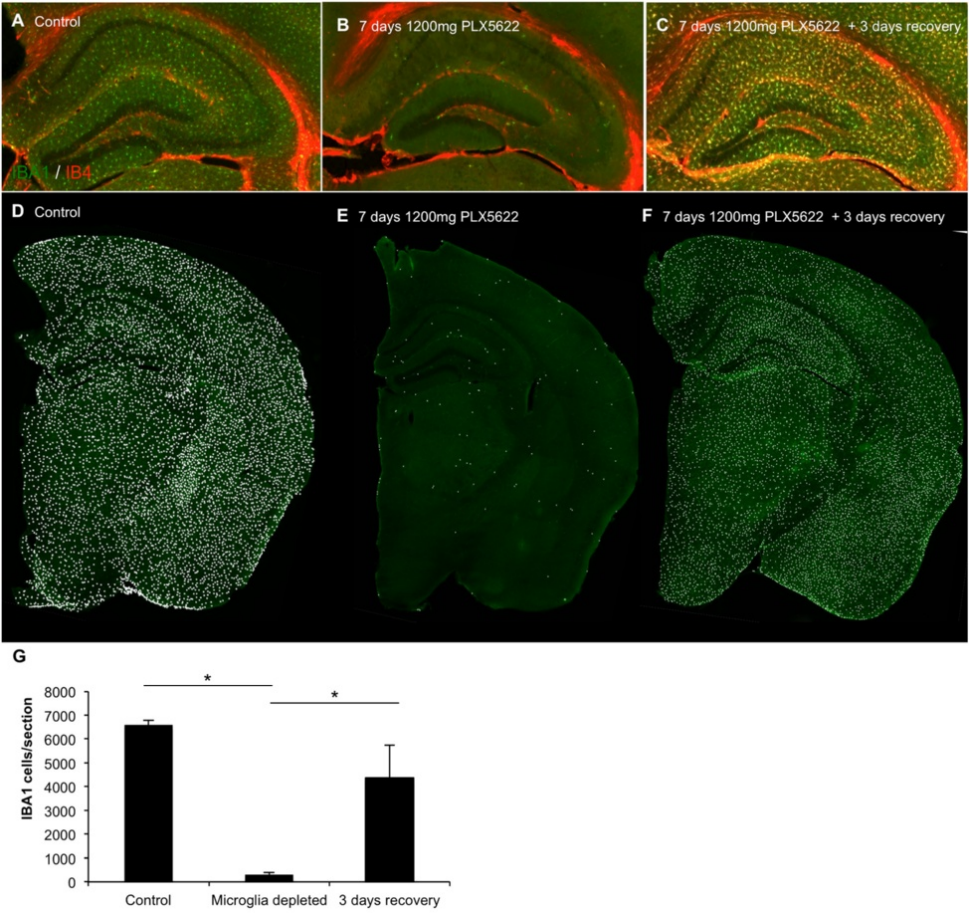
Microglial repopulation following microglial elimination with PLX5622 and subsequent PLX5622 withdrawal. Two-month-old wild-type mice were treated with 1200 mg/kg chow PLX5622 or vehicle for 7 days. The PLX5622 was then removed and the number of microglia was assessed 3 days later. **a**–**c** IBA1/IB4 staining in control, 7 days treated with PLX5622, and 7 days treated with PLX5622 with 3 days recovery, with hippocampal region shown. **d**–**f** Whole brain sections were scanned and each IBA1^+^ cell counted. Representative brain sections are shown, with a white dot superimposed over each IBA1^+^ cell as a visual aid. **g** Quantification of **d**–**f**. *Indicates significance (*p* > 0.05) via one-way ANOVA with post hoc Newman-Keuls multiple comparison test. Error bars indicate SEM

We previously showed that elimination of microglia via treatment with, and subsequent removal of, the CSF1R/c-kit inhibitor PLX3397 led to rapid repopulation of the microglial tissue. In this previous study, we found that microglial repopulation is not due to infiltration of peripheral cells and rather occurs from CNS resident non-myeloid cells [6]. To determine the involvement of c-kit in this process, we treated 2-month-old wild-type mice for 7 days with 1200 mg/kg chow PLX5622, which does not inhibit c-kit, and then withdrew PLX5622 for 3 days (*n* = 4 per group). Seven-day treatment eliminated >95 % of microglia throughout the CNS (Fig. 3a–e, quantified in G), as determined via the number of IBA1^+^ cells per brain section. Three days following drug withdrawal, repopulation of the CNS had occurred (Fig. 3f), showing that repopulation is not dependent on c-kit signaling and that elimination of microglia via CSF1R inhibition alone is sufficient to trigger the repopulation events. Repopulating cells were also detected with the lectin IB4, consistent with our prior observations [6].

### PLX5622 improves cognition in aged 3xTg-AD mice

**Fig. 4:**
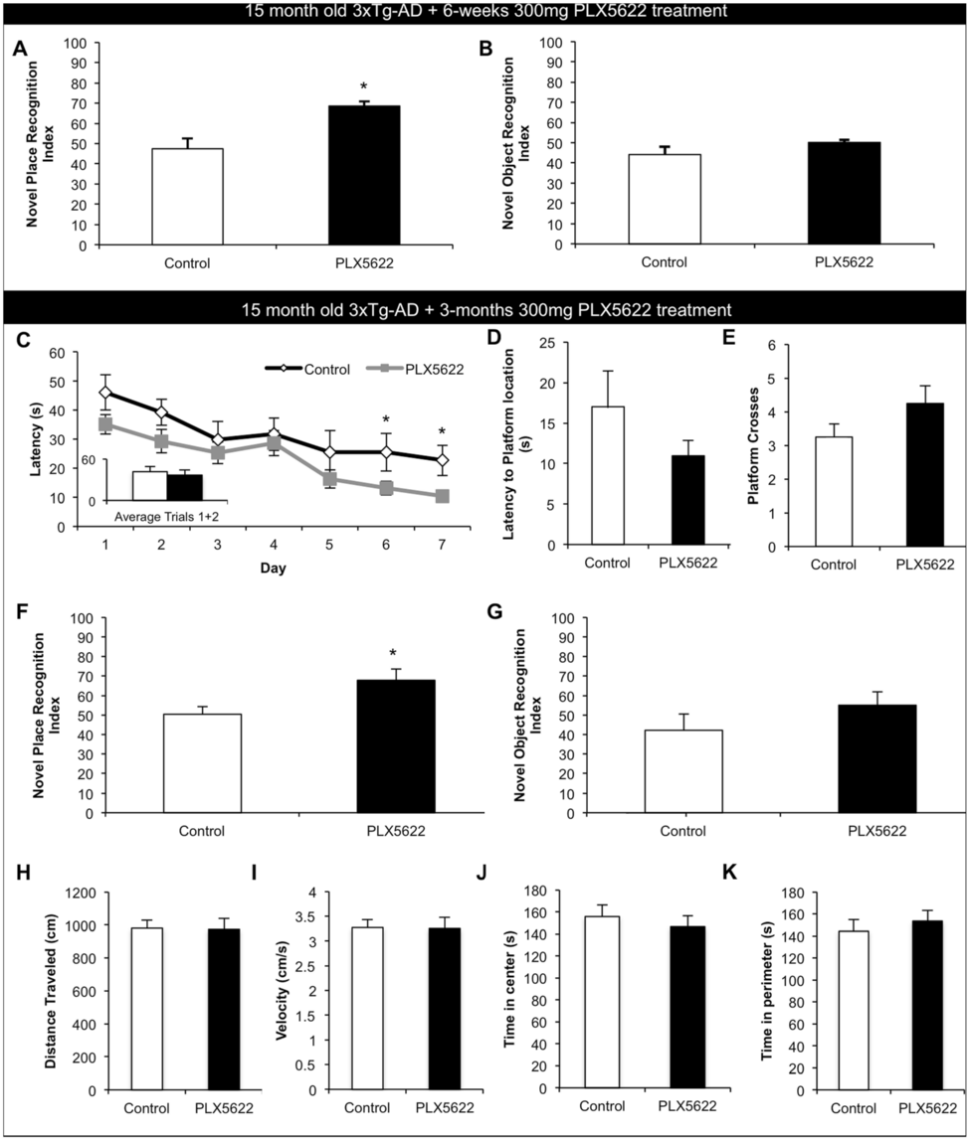
Lower doses of PLX5622 improve cognition in aged 3xTg-AD mice. Fifteen-month-old 3xTg-AD mice were treated for either 6 weeks or 3 months with vehicle or 300 mg/kg PLX5622. **a**–**b** Animals treated for 6 weeks were assessed using novel place and novel object recognition tasks. Treated mice showed a significant improvement in place recognition as compared to untreated 3xTg-AD mice, but no differences were measured between groups in novel object recognition. **c**–**k** Animals treated for 3 months were assessed using Morris water maze, novel place recognition, and novel object recognition. **c**–**e** Morris water maze. Treated mice had significantly faster escape latencies on days 6 and 7 of Morris water maze training and trended towards a faster latency to reach platform and increased platform crosses during the probe trial. **f**, **g** Treated mice showed significantly improved place recognition as compared to control mice, but no differences were shown between groups in novel object recognition. **h**–**k** No differences in open field were detected in either distance traveled (**h**), average velocity (**i**), the time spent in the center of the arena (**j**), or in the time spent in the perimeter of the arena (**k**). *Indicates significance (*p* < 0.05) by unpaired Student’s *t* test. Error bars indicate SEM

The AD brain is not only characterized by the presence of plaques and tangles but also by a chronic microglia-evoked neuroinflammatory response. Chronic neuroinflammation is harmful to the local brain environment and can be both synapto- and neurotoxic [21]. Fifteen-month-old 3xTg-AD mice, which develop both Aβ and tau pathologies as they age [22], were treated for either 6 weeks (*n* = 8 per group) or 3 months with 300 mg/kg chow PLX5662 or vehicle (*n* = 10 per group). At the end of this period, we conducted cognitive testing, followed by assessment of pathology. 3xTg-AD mice treated for 6 weeks were tested on novel place and novel object recognition, tasks that test hippocampal- and cortical-dependent memory, respectively, that rely on a rodent’s preference to explore a novel object or object location over the familiar one [23]. 3xTg-AD mice treated with PLX5622 showed significantly improved place recognition from untreated control 3xTg-AD mice (Fig. 4a), which were unable to discriminate between the objects. No differences in novel object were seen; both groups were unable to discriminate between the two objects (Fig. 4b). Having shown improvements after 6 weeks of treatment, we then set about a more extensive battery of behavioral tasks in mice treated with PLX5622 for 3 months. Mice were first tested on the Morris water maze, another hippocampal-dependent task that tests learning and memory [24]. 3xTg-AD mice treated with PLX5622 tended to have faster escape latencies than untreated mice across all days tested, with significant improvements seen on days 6 and 7 (Fig. 4c). A probe trial was conducted 24 h after the last training day, with the hidden platform removed. PLX5622-treated 3xTg-AD mice trended towards a faster latency to get to the platform location and increased platform crosses, but did not achieve significance (Fig. 4d and e). Next, mice were tested with novel place recognition and novel object recognition. Untreated 3xTg-AD mice showed no preference for either the familiar or novel place, suggesting a deficit. However, PLX5622-treated 3xTg-AD mice showed a clear preference for the novel place, indicating improvement (Fig. 4f), as with the 6-week treatment. No significant differences were seen in the novel object recognition task (Fig. 4g). The two groups of mice were also evaluated in the open-field test (Fig. 4h–k). No differences in the distance traveled or average velocity were seen, nor were there any changes in neophobic behaviors as shown by similar durations of time spent in both the center and perimeter of the arena.

### Lower-dose PLX5622 treatment partially reduces microglial numbers

**Fig. 5:**
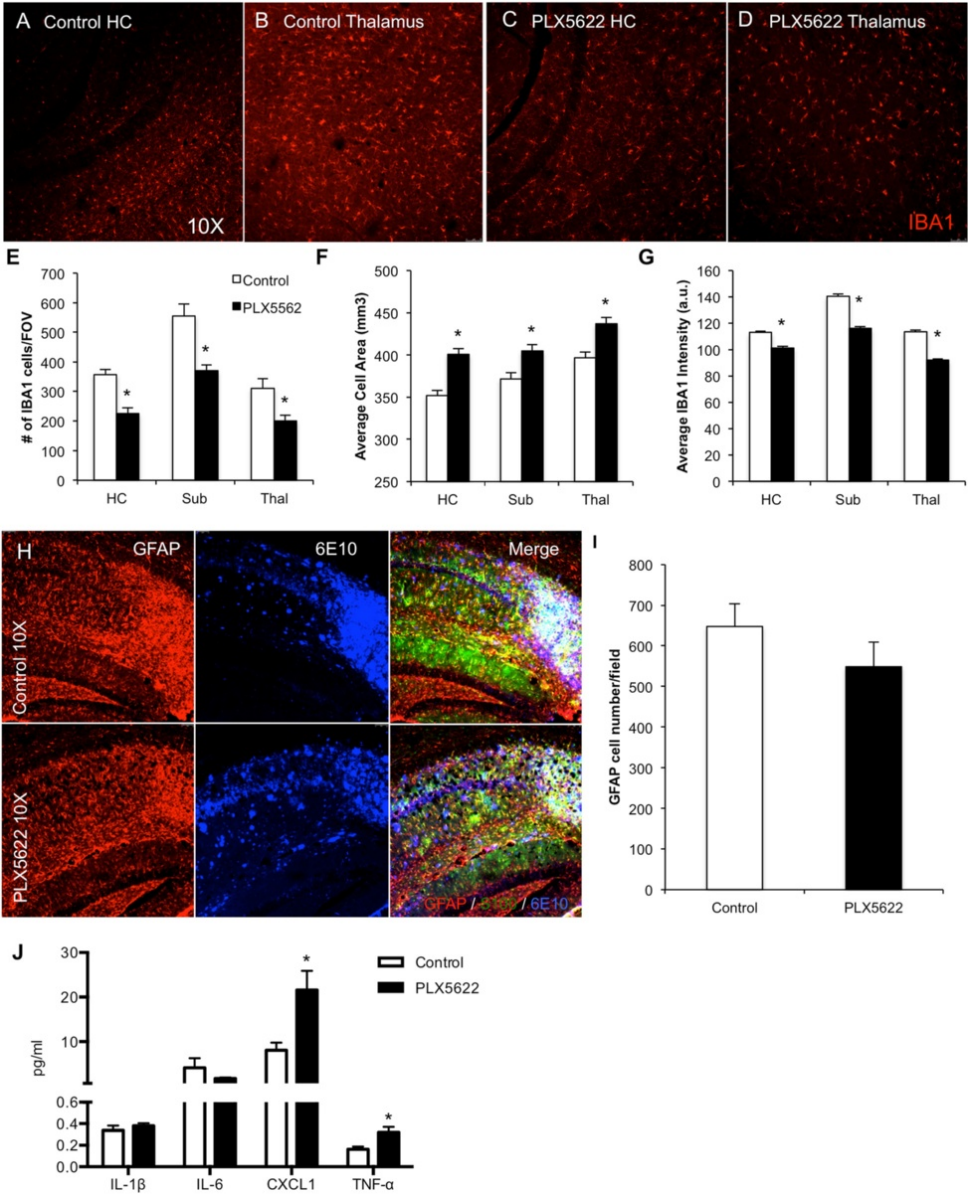
Lower-dose CSF1R inhibition partially reduces microglia numbers. The brains of 3 months 3xTg-AD-treated mice were examined for effects of PLX5622 on pathology. **a**–**d** IBA1 immunofluorescent staining was performed and representative 10× images are shown of control and treated hippocampus and thalamus. **e** IBA1^+^ cell counts revealed a reduction by 30 % in the treated groups. **f**, **g** IBA1^+^ cells in treated brains are larger but have reduced staining intensity as compared to 3xTg-AD untreated mice. **h** Immunofluorescent staining for the astrocytic markers GFAP (*red*), S100 (*green*), and plaques with 6E10 (*blue*), with the hippocampal region shown. **i** Quantification of the number of GFAP^+^ cells in the hippocampal sub-field. **j** Inflammatory profiling of whole brain homogenates shows significant increases in CXCL1 and TNFα but not Il-1β or Il-6. *Indicates significance (*p* < 0.05) by unpaired Students *t* test. Error bars indicate SEM

We next examined the brains of the 3xTg-AD mice treated for 3 months to evaluate the effects of PLX5622 on glial cells and pathology. Immunohistochemistry revealed 30 % reductions in microglial numbers in areas not adjacent to plaque loads (Fig. 5a–e), in line with the data in Fig. 1a, b, showing that we can achieve sustained targeted reductions in brainwide microglia, rather than just total elimination. Microglia in the PLX5622-treated mice had significantly larger cell sizes (Fig. 5f), in line with our previous findings [6], and also reduced IBA1 staining intensity (Fig. 5g). Immunostaining for the astrocytic markers GFAP and S100 showed robust astrogliosis associated with plaques (Fig. 5h), but PLX5622 treatment did not alter this response or the overall number of GFAP/S100^+^ cells present within the hippocampus (Fig. 5i). Inflammatory profiling using multiplex technology in soluble brain homogenates showed no changes in Il-1β or Il-6 with treatment, in line with the data obtained following LPS injections (Fig. 1e, f), but significant increases were found in CXCL1 and TNFα (Fig. 5j). Markers IFN-γ, Il-10, Il-12p70, Il-2, Il-4, and Il-5 were below detection levels.

### Lower-dose PLX5622 treatment prevents microglial association with plaques

**Fig. 6:**
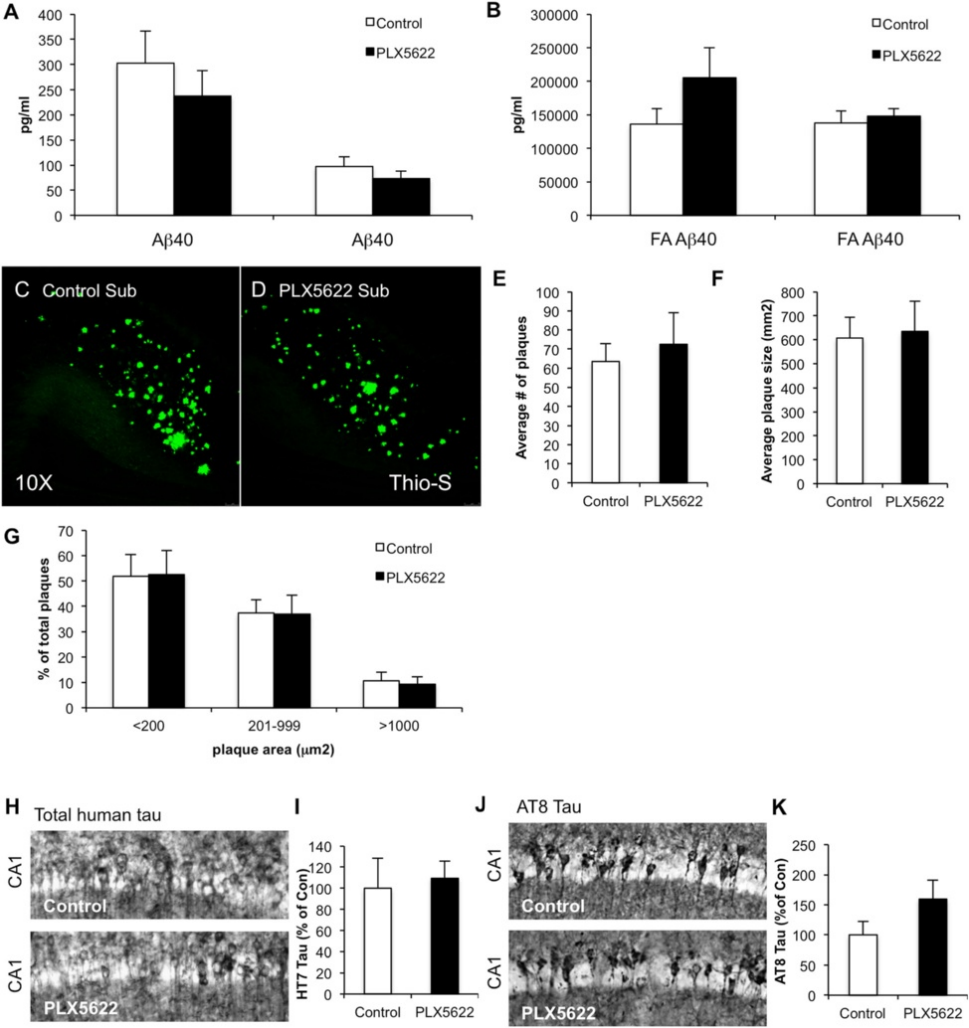
Lower-dose CSF1R inhibition does not alter Aβ or Tau levels. **a**, **b** Sandwich ELISA in soluble and insoluble fraction revealed no significant differences in Aβ40 or Aβ42 between groups. **c**, **d** Thioflavin-S staining was performed, revealing plaques in both control and treated groups. Of the subiculum, 10× images are shown. **e**–**g** Analysis of plaques revealed no significant difference between treated and untreated brains in average number of plaques, average plaque size, or distribution of large, medium, and small plaques. **h**–**k** Immunostaining for total human tau with HT7 (**h**, **i**) and AT8 tau (**j**, **k**) reveals no significant differences in tau levels with PLX5622 treatment. Error bars indicate SEM

Sandwich ELISA for Aβ40 and Aβ42 in the soluble and insoluble fractions revealed no significant differences between the PLX5622-treated group and the controls (Fig. 6a, b). Thio-S staining revealed abundant dense core plaques in all animals, particularly in the subiculum (Fig. 6c, d). Quantification of number of plaques (Fig. 6e), average plaque size (Fig. 6f), or distribution of small, medium, or large plaques (Fig. 6g), revealed no changes with treatment.

**Fig. 7:**
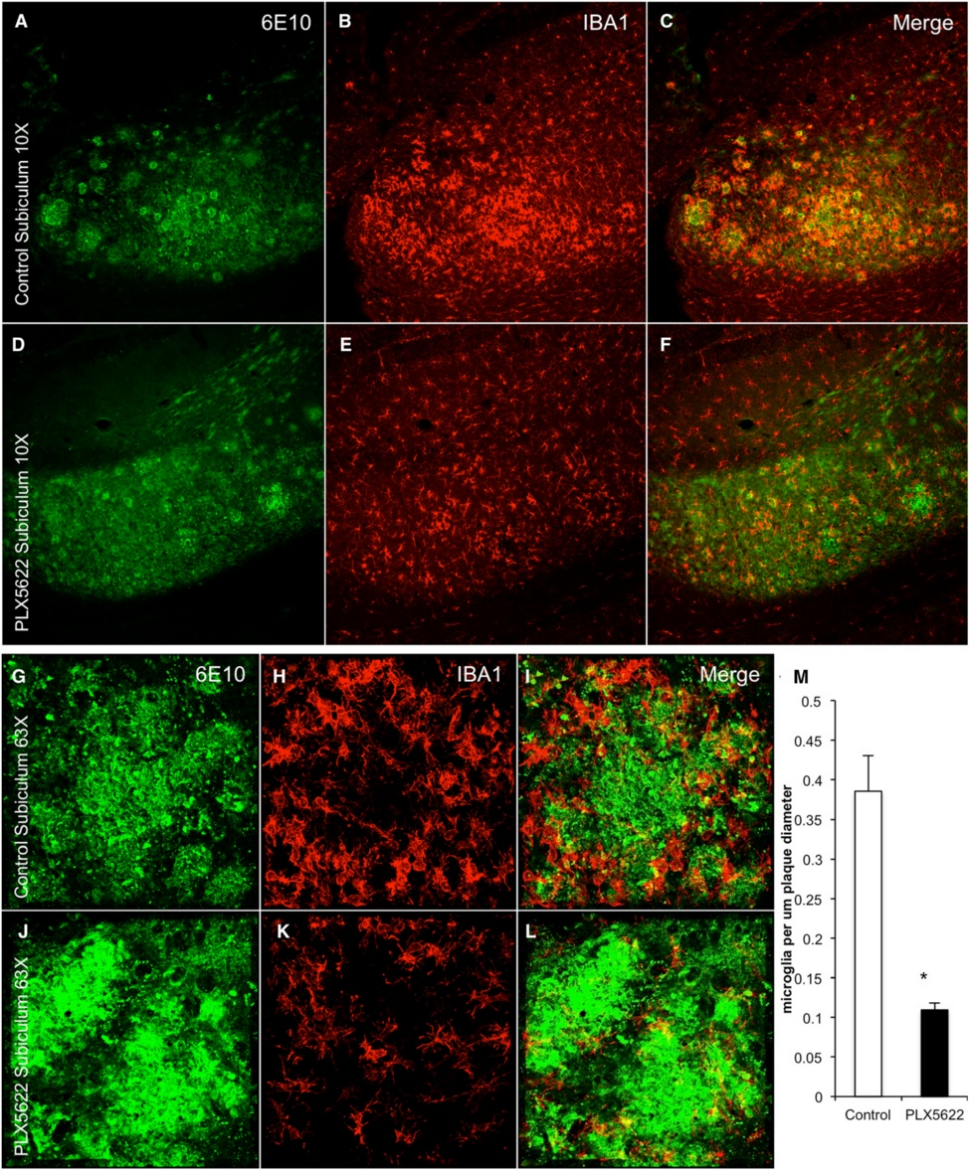
Chronic lower-dose CSF1R inhibition prevents microglia associating with plaques. Immunofluorescent staining was performed on 3 months treated and control 3xTg-AD mice for 6E10, which recognizes Aβ plaques and IBA1. **a**–**f** Representative 10× images are shown of control and treated mice. **g**–**l** Representative 63× images are shown of control and treated mice, centered on an area dense with plaques. **m** Quantification of the number of microglia associated with a plaque and normalized to plaque diameter revealed a 70 % decrease in treated animals as compared to untreated animals. *Indicates significance (*p* < 0.05) by unpaired Students *t* test. Error bars indicate SEM

However, stark differences were seen in the microglia that are associated with plaques—in vehicle-treated animals, microglia were densely packed around plaques and displayed an activated morphology (Fig. 7a–c). Mice treated with PLX5622 showed comparable plaque load, but microglia were not associated with plaques to the same extent (Fig. 7d–f), suggesting that low doses of PLX5622 do not fully eliminate microglia but alter their response to inflammatory stimuli, such as plaques. Indeed, closeup z-stacks showed a clear association of microglia with plaques in untreated 3xTg-AD mice (Fig. 7g–i) but a lack of association in PLX5622-treated mice (Fig. 7j–l). Quantification of the number of microglia associated with a plaque and normalized to plaque diameter revealed a 70 % reduction in microglia associated with plaques. To explore the possibility that this lower dose of CSF1R inhibitor could be selectively killing plaque-associated microglia, we performed immunostaining for active caspase-3, as an indicator of cell death. However, no increased microglial cell death was seen in PLX5622-treated mice in areas adjacent to plaques (data not shown). This suggests that the reduction in the number of microglia associated with plaques is due to altered microglial behavior, although it is possible that susceptible plaque-associated microglia are already dead and no further cells are going through the cell death process at this point.

3xTg-AD mice also show progressive tau pathology as they age, including somatodendritic accumulation of human tau and hyperphosphorylation [25]. Quantification of total human tau accumulation within somatodendritic compartments of CA1 neurons showed no differences with treatment, nor tau phosphorylated at S202/T205 (Fig. 6h–k).

### PLX5622 prevents chemotaxis of BV2 cells in response to Aβ-stimulated enriched media

**Fig. 8:**
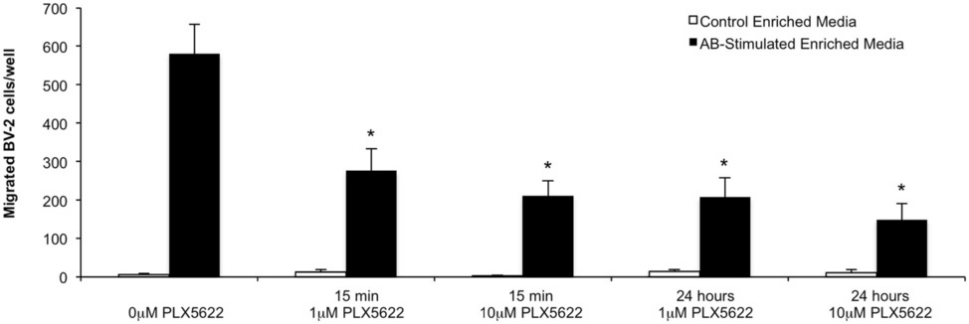
PLX5622 inhibits chemotaxis of BV-2 cells in response to Aβ-oligomer-stimulated enriched media. Chemotaxis was measured by counting migrated BV2 cells in response to Aβ-stimulated enriched media or control enriched media. BV2 cells were treated with 0-, 1-, or 10 μM PLX5622 either 15 min or 24 h before the assay was conducted. All treated cells exhibited significantly reduced cell migration in response to the Aβ-stimulated enriched media

In order to determine the mechanism behind the reduced microglial association with Aβ plaques, we conducted a chemotaxis assay on BV2 microglial cells treated with 1- or 10 μM PLX5622 (equivalent to a low- and high-dose of PLX5622, respectively) for 15 min or 24 h (*n* = 5 per group). Aβ-oligomer-stimulated enriched BV2 media was used as a chemoattractant in a ChemoTx® Chemotaxis System, and chemotaxis was measured by BV2 cell migration towards the media. All treated groups with PLX5622 showed significantly reduced cell migration to the Aβ-enriched media, indicating an impaired ability to respond to the chemoattractant signals produced by Aβ-enriched BV2 media, supporting the lack of microglial association with plaques in the treated brain (Fig. 8).

## Discussion

We previously discovered that administration of the dual CSF1R/c-kit kinase inhibitor PLX3397 led to the rapid elimination of >99 % of all microglia from the CNS within 7–21 days [6]. CSF1R knockout mice are born without microglia [7, 8], suggesting that signaling through this receptor is crucial for the development of microglia. These mice are born with developmental defects and die a few weeks after birth, making them an unsuitable model for studying microglial function. The CSF1R has two endogenous ligands—CSF1 and IL-34 [10]. Mice lacking either one of these ligands are also born with lower densities of microglia throughout the CNS [9], and diminished numbers of microglia are maintained throughout life. Thus, the CSF1R seems crucial for microglial development and also population maintenance, as well as microglial proliferation during responses to neurodegeneration [26]. As our previous study inhibited both CSF1R and c-kit, we set out to determine if inhibition of CSF1R alone was sufficient to eliminate microglia from the adult brain. Our results with the specific CSF1R inhibitor PLX5622 clearly show that inhibition of CSF1R alone is sufficient to eliminate microglia, and therefore, microglia require CSF1R signaling for their survival. Crucially, we found that lower doses of CSF1R inhibitor could lead to sustained elimination of a percentage of microglia, thus allowing us to tightly control the number of surviving microglia through different concentrations of CSF1R inhibitors. This approach may be more practical for clinical applications, where complete elimination of microglia for extended periods of time may be undesirable. Thus, we further sought to establish the effects and benefits of this paradigm in both healthy and diseased mice.

We previously demonstrated that elimination of microglia with PLX3397 had no detrimental effects on locomotion, cognition, or behavior, despite mice being depleted of microglia for up to 2 months [6]. This was an unexpected finding, as a role for microglia in synaptic sculpting [5, 27] and neuronal communication [28] is emerging. However, we confirm our prior results and show that elimination of microglia with PLX5622 leads to no discernable deficits in behavior or learning and memory in the tasks tested. Likewise, lower levels of CSF1R inhibitor treatment had no effects on behavior or learning in wild-type mice. As prolonged near-complete microglial elimination, which is achieved with the higher doses of CSF1R inhibitors, may not be translatable for the full duration of a neurodegenerative disease, lower levels of CSF1R inhibitors may offer a chronic option for the treatment of neuroinflammation. To that end, we also tested chronic treatment with the lower dose of PLX5622 in aged 3xTg-AD mice. As with wild-type mice, this lower dose resulted in sustained elimination of only ~30 % of microglia, even over a 3 month period. Strikingly, however, this treatment strongly diminished the association between microglia and plaques; untreated 3xTg-AD mice have high plaque burdens and all plaques are tightly surrounded by numerous microglia, yet treated 3xTg-AD mice have the same plaque burdens, but their microglia no longer surround them. Despite this lack of microglia associated with plaques, Aβ levels and plaque numbers and sizes were not altered, suggesting that microglia surrounding plaques are not actively restricting their growth or formation, consistent with previous findings [29].

Inflammatory status has been linked to cognitive deficits in AD patients [30], with anti-inflammatory treatments improving cognition in transgenic models of the disease [18]. Here, we found that targeting microglia with CSF1R inhibitors also led to improvements in cognition in the 3xTg-AD mice. We find that low dose treatments reduce brain microglial number by 30 %, but this does not diminish the overall levels of inflammatory markers. In line with these results, we explored the inflammatory response to LPS in wild-type mice with the same 300 mg/kg PLX5622 treatment and also found no significant effects of treatment. Moreover, we actually found increases in the levels of the typically proinflammatory markers TNFα and CXCL1 with treatment in the 3xTg-AD mice. Though seemingly counterintuitive, these results direct our attention towards other possible, non-inflammatory, mechanisms of action of PLX5622 treatment for improved behavior, potentially by acting as a “microglial shaper.” Notably, we find that this treatment prevents the association of microglia with plaques, suggesting that CSF1R inhibition alters the microglial phenotype and results in behavioral improvements. While we cannot determine the relative contributions of modest reductions in microglial numbers vs. prevention of microglial association with plaques, treatment with 300 mg/kg PLX5622 ultimately results in improved cognition. Although average cell area was increased in surviving microglia, which we have found to be a stereotypical response to CSF1R inhibition [6], we found average IBA1 staining intensity to be reduced in PLX5622-treated mice. As differential levels of IBA1 expression have also been linked to migratory function [31], the reduction in IBA1 staining intensity supports the hypothesis that microglial function is altered with 300 mg/kg PLX5622 treatment.

Of interest, recent studies investigating the effects of the AD-associated TREM2 gene on AD pathology found that heterozygous loss of one or two TREM2 alleles altered the microglial response to Aβ plaques [32–34], paralleling the effects of lower doses of PLX5622. CSF1 signaling can be regulated by TREM2 [35, 34], which could suggest that the effects of TREM2 on microglia in the AD brain may be partly mediated by the CSF1 signaling cascade. It may be that association and chemotaxis of microglia to Aβ deposits are protective in the initial stages of the disease when the microglia can help clear the aggregates from the brain, but that preventing this association at later stages alters the chronic neuroinflammatory response and becomes beneficial, as we describe here.

## Conclusions

We find that inhibition of CSF1R alone is sufficient to eliminate microglia but the level of elimination is both dose-dependent and chronically sustainable. Elimination of microglia does not impair behavior or cognition in wild-type mice. Of disease and translational relevance, lower dose inhibition of the CSF1R in 3xTg-AD mice prevents microglial association with plaques and improves cognition.
